# Mitochondria-controlled signaling mechanisms of brain protection in hypoxia

**DOI:** 10.3389/fnins.2015.00320

**Published:** 2015-10-01

**Authors:** Ludmila D. Lukyanova, Yulia I. Kirova

**Affiliations:** Laboratory for Bioenergetics and Hypoxia, Institute of General Pathology and PathophysiologyMoscow, Russia

**Keywords:** hypoxia, reprogramming of respiratory chain function, HIF-1a, GPR91, complexes I and II

## Abstract

The article is focused on the role of the cell bioenergetic apparatus, mitochondria, involved in development of immediate and delayed molecular mechanisms for adaptation to hypoxic stress in brain cortex. Hypoxia induces reprogramming of respiratory chain function and switching from oxidation of NAD-related substrates (complex I) to succinate oxidation (complex II). Transient, reversible, compensatory activation of respiratory chain complex II is a major mechanism of immediate adaptation to hypoxia necessary for (1) succinate-related energy synthesis in the conditions of oxygen deficiency and formation of urgent resistance in the body; (2) succinate-related stabilization of HIF-1α and initiation of its transcriptional activity related with formation of long-term adaptation; (3) succinate-related activation of the succinate-specific receptor, GPR91. This mechanism participates in at least four critical regulatory functions: (1) sensor function related with changes in kinetic properties of complex I and complex II in response to a gradual decrease in ambient oxygen concentration; this function is designed for selection of the most efficient pathway for energy substrate oxidation in hypoxia; (2) compensatory function focused on formation of immediate adaptive responses to hypoxia and hypoxic resistance of the body; (3) transcriptional function focused on activated synthesis of HIF-1 and the genes providing long-term adaptation to low pO_2_; (4) receptor function, which reflects participation of mitochondria in the intercellular signaling system via the succinate-dependent receptor, GPR91. In all cases, the desired result is achieved by activation of the succinate-dependent oxidation pathway, which allows considering succinate as a signaling molecule. Patterns of mitochondria-controlled activation of GPR-91- and HIF-1-dependent reaction were considered, and a possibility of their participation in cellular-intercellular-systemic interactions in hypoxia and adaptation was proved.

## Mitochondrial signaling function and the body vital activity

Oxygen dependence and capability for maintaining the oxygen homeostasis are the properties of all higher organisms whose vital activity is related with aerobic energy production due to functioning of the respiratory chain in the inner mitochondrial membrane. Inhaled air reflects the state and oxygen demand of mitochondria (Balaban, 1977; Chandel and Schumacker, 2000; Duchen et al., 2003; Duchen, 2004; Gnaiger, 2005; Das, 2006; Devin and Rigoulet, 2007; Kann and Kovacs, 2007; Wheaton and Chandel, 2011).

In mammals, up to 98% of body consumed oxygen is related with oxidative phosphorylation, a process occurring in the respiratory chain, where O_2_ is the final acceptor of electrons delivered by NADH and flavoproteins (Silver and Erecinska, 1998). The electromotive force generated in this process is used for formation of membrane potential and ATP synthesis, which are required for various energy-dependent reactions responsible for maintaining the cell vitality.

Mitochondria, being oxygen-dependent, are a target for hypoxia/ischemia. Shortage of cell environmental oxygen decreases aerobic synthesis and content of high-energy compounds (ATP, CP) and reduces the membrane potential required for maintaining energy demands and the osmotic balance. These factors, in turn, lead to depression of multiple energy-dependent reactions involved in ion transport, electrogenic and receptor cell function, muscle contractions, respiration, etc. The most typical disorders are membrane depolarization, uncontrolled Ca^2+^ entry through voltage-dependent Ca^2+^ channels, activation of Ca^2+^ -dependent phospholipases and proteases, uncontrolled swelling of cells, hydrolysis of most cell components, and, finally, necrosis. Thereby, mitochondrial dysfunction induced by hypoxia may initiate development of various abnormalities and even cause fatality (Acker, 1994; Lukyanova, 1997, 2014; Rumsey et al., 1999; Wenger, 2000; Peers and Kemp, 2001; Lutz and Prentis, 2002; Michiels, 2004; Gnaiger, 2005; Heerlein et al., 2005; Larsen et al., 2006; Seppet et al., 2009; Wheaton and Chandel, 2011; Lukyanova et al., 2013).

Brain is one of the most oxygen-dependent organs (Siesjo, 1978; Nicholls and Budd, 2000; Bickler and Donohoe, 2002; Neubauer and Sunderram, 2004; Kann and Kovacs, 2007). Brain uses up to 20% of consumed oxygen, although the brain weight constitutes only 2% of body weight (Siesjo, 1978; Silver and Erecinska, 1998). Brain is characterized by high expenditure of energy and low energy reserve. Furthermore, maintenance of energy-dependent process takes 80% of ATP produced in neuronal mitochondria. For this reason, the brain is extremely sensitive to hypoxia, particularly specific brain regions, such as the cortex and hippocampus. All this makes essential the insight into functioning of the brain mitochondrial apparatus in the conditions of insufficient oxygen supply.

According to current concepts, mitochondria are an evolutionary product of endosymbiosis, a process that has occurred 2–4 billion years ago. This process involved fusion of two akaryocytes, an anaerobic bacterial cell, the “host,” and a proteobacterium symbiont, which possessed a respiratory apparatus including elements of the tricarbonic acid cycle (TAC), a respiratory chain, and oxidative phosphorylation (Margulis, 1981; Alberts et al., 1989; Gray et al., 1999, 2001; Martin et al., 2001; Henze and Martin, 2003; Duchen, 2004; Gray, 2012). In this symbiosis, the future symbiont mitochondrion (promitochondrion) provided the host cell with energy, which was produced by a very economical, aerobic way, while the host cell, in turn, synthesized metabolites necessary for the mitochondrion. During the development of endosymbiotic interrelations, proteobacteria transferred many genes to the host cell nucleus, which had formed due to the increased energy efficiency. This process resulted in simplification of the promitochondrion endosymbiont, which evolved from an independent microorganism into a cell organelle. In this way, the symbiont cells turned into mitochondria whereas host cells turned into eukaryocytes. Due to the endosymbiotic relations, the pro-eukaryocyte has received not only energetic advantages but also a chance for survival in the conditions of gradual oxygen accumulation in the atmosphere, which had initially contained very little oxygen, less than 0.1%. Before oxygen enrichment of the atmosphere, all forms of life that existed in the biosphere of that time, were anaerobic (Margulis, 1981; Alberts et al., 1989; Gray et al., 1999, 2001; Martin et al., 2001; Henze and Martin, 2003; Duchen, 2004; Gray, 2012).

Current mitochondria are unique cytoplasmic organelles characteristic of eukaryotes, i.e., the organisms with cells containing formed, membrane-confined nuclei. Due to their origin, mitochondria possess three distinctive features of structural organization, which differ it from all other intracellular organelles: (1) presence of respiratory chain enzymes designed for aerobic energy production and arranged into four complexes. These complexes are capable for uniting into supercomplexes (respirasomes), which stabilizes the electron transport chain performance, increases the electron transport velocity, may involve substrate channeling, and restricts generation of reactive oxygen species as a by-product (Acín-Pérez et al., 2004; Schagger et al., 2004; McKenzie et al., 2006; Schäfer et al., 2006; Starkov, 2008); (2) presence of its own genome, which allows renewing components of respiratory chain complexes, the most functionally important proteins, independently on the nucleus genome in the conditions of intensive functional loads; and (3) capability for motion, including division, fusion, and intracellular traveling (Kuznetsov et al., 1994, 2004; Gray et al., 1999, 2001; Kaasik et al., 2001; Martin et al., 2001; Henze and Martin, 2003; Chen and Chan, 2005; Kuznetsov and Margreiter, 2009; Gray, 2012; Van der Bliek et al., 2013). Due to the latter property known in scientific literature as “mitochondrial dynamics,” mitochondria can form functional complexes with the endoplasmic reticulum and cytoskeletal structures (*intracellular functional energetic units, mitochondrial reticulum*). These complexes provide energy metabolism in local energy-transportation networks. In these complexes, ADP diffusion is facilitated, and a system of channels for metabolite exchange is available. The capability of mitochondria for structural remodeling and metabolic reprogramming is a basis mechanism inseparably linked with mitochondrial energy production, which provides interaction of mitochondria with each other and with other cell structures and systems. Therefore, in the process of evolution, the mitochondrial major function of aerobic energy production has allowed mitochondria to become involved in *regulation of various physiological functions* by providing energy to most of intracellular processes necessary for the body vital activity (Kaasik et al., 2001; Appaix et al., 2003; Chen and Chan, 2005, 2009; Schäfer et al., 2006; Kuznetsov and Margreiter, 2009; Van der Bliek et al., 2013).

Mitochondria actively participate in *cell metabolism*. They contain many key, limiting enzymes for fatty acid oxidation, steroid biosynthesis, heme synthesis, insulin secretion in β-cells (MacDonald et al., 2005) and gastric juice secretion (Kennedy and Lehninger, 1949; Gnaiger et al., 2000; Duchen et al., 2003; Duchen, 2004; Gnaiger, 2005). Mitochondria participate in regulation of cell redox potential (Meister, 1995) and protein import (Wiedemann et al., 2004). In addition, mitochondria contribute to intracellular signaling and regulation with a central role in keeping homeostatic cell ionic composition (Chandel, 1990; Chandel and Schumacker, 2000; Butow and Avadhani, 2004; Darley-Usmar, 2004). A system of regulatory interaction related with Ca^2+^ and K^+^- metabolism exists between mitochondria and the endoplasmic reticulum (Gunter and Gunter, 1994; Hansford and Zorov, 1998; Liu et al., 1998; Rizzuto et al., 1998; Fiermonte, 1999; Da Silva et al., 2003).

A system exists that is connected to Ca^2+^ metabolism and involves regulatory interactions between mitochondria and the endoplasmic reticulum as well as between activation of G-coupled surface receptors, the phosphatidyl-inositol-3-kinase (PI3K) cascade, eNOS, guanylyl cyclase, and protein kinase G (PKG), and performance of the mitochondrial ATP-sensitive potassium (mitoK_ATP_) channel (Wang and Semenza, 1993; Pek and Lutz, 1998; Bernaudin et al., 2004; He et al., 2004; Murphy, 2004; Fan et al., 2010). Activated PKG opens the mitoK_ATP_ channel, which results in increased ROS production followed by activation of other kinases. In this system, PKG is the terminal cytosolic component of the terminal signaling pathway transmitting the cardioprotective signal from the cytosol to the inner mitochondrial membrane through the protein kinase C (PKC)-dependent pathway (Wang and Semenza, 1993; Zhu and Bunn, 1999; Stroka et al., 2001; Semenza, 2002; Bruick, 2003; Butow and Avadhani, 2004; Murphy, 2004). In addition, mitochondria participate in cell-to-cell interactions and systemic regulation (Zhu and Bunn, 1999; Chandel and Schumacker, 2000; Nicholls and Budd, 2000; Nishimura et al., 2001; Brunk and Terman, 2002; Butow and Avadhani, 2004; Kuznetsov et al., 2004; Michiels, 2004; Felty and Roy, 2005; MacDonald et al., 2005; Lukyanova et al., 2008a, 2011). Mitochondria are the major regulators of the *oxygen homeostasis in the body*. At the *systemic* level, mitochondria determine the concentration gradient for oxygen delivered from the environment to the cell and represent the final step of interaction with molecular oxygen (Brunk and Terman, 2002; Lukyanova et al., 2008a, 2011). Due to this function, which determines both viability and vital activity of aerobic organisms, evolution has created very sophisticated physiological systems for oxygen delivery to mitochondria and maintenance of optimum oxygenation in cells (act of breathing; pulmonary system of oxygen transportation; cardiovascular circulatory system; blood mass-transfer system; red cells; and hemoglobin) (Zhu and Bunn, 1999; Nicholls and Budd, 2000; Prabhakar and Overholt, 2000; Di Lisa and Ziegler, 2001; Peers and Kemp, 2001; Brunk and Terman, 2002; Voos and Rotgers, 2002; Da Silva et al., 2003; Duchen, 2004; Kuznetsov et al., 2004; Michiels, 2004; Devin and Rigoulet, 2007).

Organization of the digestion system, including consumption and subsequent stepwise enzymic processing of the food, is also dictated primarily by the need for substrate supply to reactions of mitochondrial oxidation and oxidative phosphorylation (Michiels, 2004). The signaling function of mitochondria is related with such processes as growth (Felty and Roy, 2005), aging (McCarter, 1995; Brunk and Terman, 2002), response to temperature (thermogenesis), apoptosis (Kroemer et al., 1998; Nishimura et al., 2001), insulin secretion in β-cells (MacDonald et al., 2005), and formation of adaptive responses (Bruick, 2003).

Mitochondria provide energy for physiological functions involved in the body vital activity. These functions include, first of all, maintenance of ion gradients in excitable tissues; accumulation of vesicular secret; maintenance of hormonal and neurotransmitter functions; contractility of the heart and vascular, pulmonary and gastrointestinal smooth muscles; fertilization and embryogenesis; and adaptation to stresses (Zhu and Bunn, 1999; Nicholls and Budd, 2000; Di Lisa and Ziegler, 2001; Peers and Kemp, 2001; Voos and Rotgers, 2002; Da Silva et al., 2003; Duchen, 2004; Kuznetsov et al., 2004; Devin and Rigoulet, 2007; Seppet et al., 2009).

Therefore, the mitochondrial respiratory chain in involved not only in the intracellular signaling, but also transmembrane and intercellular signaling. Mitochondria themselves function as active signaling organelles, which contribute to information transmission in various intracellular signaling pathways, and play a key role in most important regulatory physiological processes.

## Regulatory role of mitochondria in hypoxia

The body response to hypoxia includes various adaptive reactions, which facilitate elimination of functional and metabolic disorders typical for hypoxia and focus primarily on preservation of the mitochondrial function. In this process, two types of mechanisms are used: (a) an urgent compensatory mechanism designed for mobilization of immediate responses to prevent or eliminate the disorders induced by acute hypoxia; these responses provide fast recovery of the body in the posthypoxic period; and (b) delayed mechanisms of adaptation to hypoxia, which develop within a longer period and enhance non-specific resistance to oxygen shortage. These mechanisms are based on regulatory reprogramming of mitochondrial complex function.

The mitochondrial respiratory chain consists of a series of electron carriers that function as redox pairs and that are mainly prosthetic groups of integral proteins. There are four electron transfer or respiratory complexes (complexes I–IV), each capable of catalyzing electron transfer in a partial reaction of the respiratory chain. Complex I (NADH-ubiquinone oxidoreductase) (C-I) is a multisubunit complex that possesses the NADH-ubiquinone oxidoreductase activity. This largest component of the respiratory chain consists of at least 46 protein subunits (46–49) (Grivennikova et al., 2001). Seven of which are encoded by the human mitochondrial genome. Additionally, C-I contains stably bound cofactors, including flavin mononucleotide and eight iron-sulfur clusters. C-II (succinate-ubiquinone oxidoreductase) mediates the succinate dehydrogenase (SDH) activity. C-III is ubiquinol-cytochrome c reductase, while C-IV displays the cytochrome c oxidase activity. The electrons resulting from the oxidative process are transferred from C-I and C-II to C-III through ubiquinone and from C-III to C-IV through cytochrome ***c*** as shuttles. Electron transfer through complexes I, III, and IV is coupled to proton pumping, creating the electrochemical gradient used by complex V (ATP synthase) to synthesize ATP from ADP and in organic phosphate (Genova et al., 1995; Brandt, 1997; Briére et al., 2004; Schagger et al., 2004; Schäfer et al., 2006; Lenaz and Genova, 2007).

In the normoxic conditions, ***r***espiratory complexes are assembled into supercomplexes. Complexes I, III, and IV exist together as supramolecular assemblies called respirasomes. The likely functions of the supercomplexes are facilitating the electrons transfer between complexes and minimizing the risk of releasing electrons. The assembly of a respirasome might have advantages in substrate channeling of quinones and/or cyt *c*, sequestration of reactive intermediates, and stabilization of individual complexes. In humans, it was shown that supercomplexes are essential for C-I stability (Acín-Pérez et al., 2004; Schagger et al., 2004; Althoff et al., 2011; Chen et al., 2012; Moreno-Lastres et al., 2012; Winge, 2012). In contrast to the organized electron flow by respiratory supercomplexes, mitochondrial C-II (SDH) can function as an independent enzyme whose activity is limited only by substrate availability (Hawkins et al., 2010).

**Figure 1:**
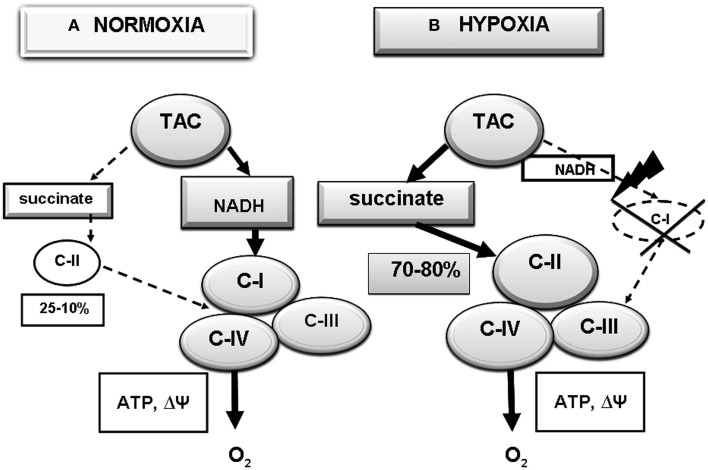
**Reprogramming of the respiratory chain function and switching from oxidation of NAD-related substrates (Complex I, C-I) in normoxia (A) to succinate oxidation (C-II) in hypoxia (B). (A)** Normoxia; high activity of C-I (70–80% of mitochondrial respiration), low activity of C-II (25–10% of mitochondrial respiration); **(B)** hypoxia; depression of C-I, activation of C- II (90–75% of mitochondrial respiration).

In normoxia, performance of the respiratory chain usually depends on oxidation of NAD-dependent substrates, the major supplier of reduction equivalents to the respiratory chain through C-I (Figure 1A). Contribution of this pathway, as estimated by oxygen consumption, predominates in intact cells. In brain, this contribution may reach 90% relative to the total mitochondria oxygen consumption (Lukyanova, 1997, 2014; Lukyanova et al., 2009a, 2013). Nevertheless, in these conditions, a part of mitochondrial respiration is related with the activity of an alternative pathway of electron transport through C-II and oxidation of succinate, a TAC cycle product found in relatively low concentrations (0.2–0.4 mM) in the normoxic mitochondrial matrix (Hems and Brosnan, 1970; Komaromy-Hiller et al., 1997). In normoxia, the ratio of the two oxidation pathways (NAD-dependent and succinateoxidase) depends primarily on properties of the major C-I and C-II enzymes, NADH-ubiquinone oxidoreductase and SDH, respectively. Their kinetic characteristics are tissue-specific and differ in animals with different hypoxia tolerance (Lukyanova, 1988, 1997, 2005; Dudchenko et al., 1993; Dudchenko and Luk'yanova, 1995, 1996; Lukyanova and Dudchenko, 1999; Lukyanova et al., 2008b, 2009a,b).

Hypoxia induces regulatory reprogramming of the respiratory chain, including reversible suppression of the C-1 electron transport function and compensatory activation of C-II Lukyanova, 1997; Lukyanova et al., 2008a, 2013). The dissociation of C-I from the large supercomplexes occurs under the hypoxic conditions, when succinate accumulates as a substrate for C-II (Figure 1B) (Sanborn et al., 1979; Acín-Pérez et al., 2004; Althoff et al., 2011; Chen et al., 2012; Moreno-Lastres et al., 2012; Winge, 2012).

Hypoxia is associated with activation of succinate dehydrogenase and succinate oxidation and with increased contribution of the latter to respiration and energy production (Acín-Pérez et al., 2004; Lenaz and Genova, 2007; Althoff et al., 2011; Chen et al., 2012; Moreno-Lastres et al., 2012). The contribution of succinate oxidase oxidation may reach 70–80% (Lukyanova et al., 2008a, 2009a). Under these conditions, mitochondrial C-II can function as an independent enzyme whose activity is limited only by the substrate availability. The C-II-driven electron flow is the primary way of mitochondrial membrane polarization under the hypoxic conditions and that lack of the C- II substrate succinate resulted in reversible membrane potential loss that could be restored rapidly by succinate supplementation (Nowak et al., 2008; Hawkins et al., 2010). The inhibition of mitochondrial C-II also leads to mitochondrial depolarization and mimics hypoxia in cells. The effect of reversible inactivation of the C-I electron transport function in hypoxia is the first stage in the development of hypoxia-induced mitochondrial disorders, which correlate with functional and metabolic phase changes observed at the systemic level (Lukyanova, 1997; Lukyanova et al., 2009b, 2013; Lukyanova, 2014).

At present time, much experimental evidence is available in support of hypoxia-induced disorders in the C-I electron transport function. These disorders persist and even increase in the early posthypoxic period (first 30 min to 2 h of reoxygenation) and are known as *mitochondrial dysfunction* (Rouslin and Millard, 1980; Lukyanova, 1988; Genova et al., 1995; Pitkanen and Robinson, 1996; Robinson, 1998; Chávez et al., 2000; Kunz et al., 2000; Weinberg et al., 2000; Maklashinas et al., 2002; Da Silva et al., 2003; Feldkamp et al., 2004; Sadek et al., 2004; Regard et al., 2008; Lukyanova et al., 2009a).

Also, much evidence exists for a special role of succinate in tissue oxygen metabolism at early hypoxia. Thus, tissue and blood content of succinate was shown to increase by an order of magnitude up to 4–7 mM already in the first 30 min of hypoxia, and to continue growing through early reoxygenation, which allowed some researchers to consider succinate a *hypoxia marker molecule* (Hems and Brosnan, 1970; Hochachka and Dressendorfer, 1976; Taegmeyer, 1978; Hohl et al., 1987; Komaromy-Hiller et al., 1997; Hochachka and Somero, 2001; Kushnir et al., 2001).

The activation of succinate oxidase oxidation in these conditions should be regarded *as an evolutionarily formed, urgent, protective, regulatory, and compensatory mechanism*, which occurs in most tissues *under any form of oxygen shortage* and provides preservation of the aerobic energy production during early disorders of the oxygen homeostasis (Lukyanova, 1997; Lukyanova et al., 2009a, 2013). If this switch does not happen, the uncompensated hypoxic dysfunction of C-I results in acute de-energization (decreased membrane potential; loss of ATP and changes in the adenine nucleotide pool; and respiration disorders due to oxidation of NAD-dependent substrates, the electron donators for C-I) (Dudchenko et al., 1993; Dudchenko and Luk'yanova, 1996; Lukyanova, 1997, 2005; Lukyanova and Dudchenko, 1999; Lukyanova, 2005; Lukyanova et al., 2009a).

All this taken together precedes changes in other functional and metabolic parameters that control the cell vital activity, including condensation of the mitochondrial matrix; disturbed calcium and potassium homeostasis; disordered expression of the mitochondrial genome; generation of reactive oxygen species; loss of CoQ; exit of cytochrome c to the intermembrane space; apoptosis; and impaired capability of cells for adaptation to low pO_2_. These processes are associated with an increased lactate/pyruvate ratio; changes in the cell redox potential; metabolic acidosis; and impairment of various energy-dependent processes (such as the electrogenic function of electro-excitable cells and anabolic processes, including urea synthesis, phase II biotransformation reactions, etc.

## Features of respiratory chain reprogramming in hypoxic brain cortex

The described above hypoxia-induced changes in the performance of respiratory chain are universal and occur in all tissues, although they are tissue-specific. This process is particularly important for the brain, since the major energetic substrate of oxidation in brain is glucose, which is metabolized to pyruvate in the process of glycolysis. In the aerobic conditions, pyruvate is oxidized in TCA cycle reactions in the NAD-dependent pathway. In the normoxic brain cortex (BC), this oxidation pathway may use up to 90% of consumed oxygen (Lukyanova, 1997). However it should be taken into account that pheno- and genotypic features of the body response to hypoxia are critically important^1^. Furthermore, individual differences in the response to hypoxia manifest themselves not only at the systemic level but also at the cellular and subcellular levels.

Thus, processes of oxidative phosphorylation were shown to be fundamentally different in BC mitochondria of intact hypoxia high-resistant (HR) and low-resistant (LR) rats. In BC of normoxic LR, the baseline similarity of oxidative phosphorylation efficiency is due to a higher rate of phosphorylating respiration, which indicates less economy of this process at baseline. In addition, during oxidation of NAD-dependent substrates by LR BC mitochondria, the velocity of electron transport in the respiratory chain is maximum and may exhaust its reserve capacity, should the functional load increase; this is not observed in the brain of HR animals (Luk'yanova et al., 1991; Dudchenko et al., 1993; Lukyanova et al., 2009a,c).

Differences in the oxidative capacity of the NAD-dependent substrate region in the BC respiratory chain of HR and LR animals reflect peculiarities of kinetic characteristics for some of respiratory chain enzymes. Thus, in the normoxic BC of LR rats, maximum activity and K_M_ values (NADH) of NADH-cytochrome c reductase, the main C-I enzyme, are significantly lower than in the BC of HR rats (Dudchenko et al., 1993; Dudchenko and Luk'yanova, 1995, 1996; Lukyanova, 1997, 2005, 2014; Lukyanova et al., 2013). Similar, though less pronounced differences in values of kinetic parameters are typical for cytochrome oxidase in BC of HR and LR animals. Therefore, in the LR BC, these enzymes faster become saturated with the enzyme-specific substrates (NADH or cyt-*c*) and slower oxidize them, which may result in lower activity and faster inactivation of C-I (Dudchenko and Luk'yanova, 1995, 1996; Luk'yanova et al., 1995).

The differences in kinetic parameters of respiratory chain enzymes remain also in oxygen-deficient BC of HR and LR animals. For example, in the neocortex of LR rats, any hypoxia exposure induced opposite changes in K_M_ values of both complex enzymes—increased NADH-ubiquinone oxidoreductase (C-I) K_M_ and decreased SDH K_M_ (C-II). This process reflects reduced and increased efficiency of the enzyme in the first and the second instances, respectively. For this reason, the suppression of BC C-I activity occurs earlier and is more marked in LR rats than in HR rats. Therefore, in different forms of hypoxic exposure, the switching of oxidation pathways for respiratory chain substrates from the NAD-dependent pathway to the succinate pathway is due to kinetic reasons (Luk'yanova et al., 1995). The existence of different kinetic properties of respiratory chain enzymes in HR and LR rat BC, a target for hypoxia, suggests that this phenomenon is genetically predetermined, and the energy metabolism is involved as a major factor determining formation of individual resistance.

These features of C-I and C-II responses to hypoxia correlate with changes in ATP content. Thus, evaluating the dependence of intracellular ATP level on pO_2_ showed that a decrease in ATP concentration in the LR rat BC began at higher pO_2_ values and was much more pronounced than in HR rats (Dudchenko et al., 1993; Dudchenko and Luk'yanova, 1995; Lukyanova and Dudchenko, 1999). The hypoxic reprogramming of respiratory chain substrate region not only prevents or alleviates disorders of ATP synthesis and normalizes parameters of the adenylate pool but also beneficially influences vital functions, stabilizes and normalizes pH, eliminates acidosis typical for hypoxia (Maevsky et al., 2001), and enhances resistance to the oxygen shortage (Figure 1) (Taegmeyer, 1978; Weinberg et al., 2000; Lukyanova, 2005; Lukyanova et al., 2013; Lukyanova, 2014). This process occurs rather rapidly. Changes in kinetic parameters of C-I and C-II major enzymes were observed already at 30 min of various hypoxic exposures (Lukyanova et al., 2008b).

Analyzing effects of different hypoxic regimens on the respiratory chain substrate region showed that regulatory rearrangements of the respiratory chain performance were qualitatively similar in all studied instances. These rearrangements are aimed at activation of the succinate oxidase pathway (C-II), which is energetically more efficient in the hypoxic conditions (Hawkins et al., 2010). When changes in the kinetic parameters of succinate oxidase oxidation were minor or absent, the development of resistance was difficult (Lukyanova et al., 2009a).

Qualitatively different changes in kinetic properties of BC mitochondrial enzymes occur in long-term hypoxic exposures, which provide formation of delayed adaptation (lengthy stay in mountains, hypoxia courses in different regimens). An undoubted sign of adaptation to high altitude hypoxia is increased mitochondrial mass in tissues. The increase in mitochondrial mass during long-term adaptation to hypoxia correlated with activated synthesis of nucleic acids and mitochondrial proteins to provide potentiation of the mitochondrial apparatus. The increase in brain total protein was associated with simultaneous increases in cytochromes, including cytochrome *aa*_3_, which changed in the same way in BC of both HR and LR animals. The cytochrome *aa* content per unit tissue was increased and correlated with the increase in mitochondrial mass and was decreased per unit mitochondrial protein (Dudchenko and Luk'yanova, 1996; Lukyanova and Dudchenko, 1999), which indicated a decreased amount of respiratory transporters in the respiratory chain cytochrome region. Furthermore, the decrease in oxidation rate of NAD-dependent substrates was greater in the LR rat brain than in HR rats.

However, the phosphorylation efficiency estimated by the ATP/O ratio was higher after than before long-term adaptation to hypoxia (Luk'yanova et al., 1990, 1991; Lukyanova et al., 2009b). The increased efficiency of NAD-dependent substrate oxidation together with the reduced electron transport function and the increased amount of mitochondria suggest economization of the energy production process in the brain of adapted rats. Furthermore, the velocity of electron transport in the respiratory chain was no longer limit as it had been before the adaptation, and a “respiratory activity reserve” appeared. In contrast, the “physiological range of respiratory activity” decreased. All these processes were more pronounced in the BC of LR rats. Taken together, these data suggest that long-term adaptation to hypoxia restored the leading role of NAD-dependent oxidation in the energy metabolism. Therefore, preserving high activity of specifically these pathways is essential for development of individual brain resistance to oxygen shortage. On the contrary, the participation of succinate oxidase pathway in energy metabolism during long-term adaptation to hypoxia gradually declined, particularly in the brain of LR animals, and the use of this pathway as a compensatory mechanism became restricted (Lukyanova and Dudchenko, 1999; Lukyanova, 2005).

Kinetic properties of mitochondrial enzymes also changed in the process of long-term adaptation to hypoxia. In the BC of LR rats adapted to hypoxia, Vmax values for NADH cytochrome *c* reductase and cytochrome oxidase 1.5–2.5 times increased while K_M_ (cyt *c*) values decreased, and the Vmax values approached or exceeded those for HR rat BC. In other words, in the LR BC, activities of these enzymes increased during adaptation to hypoxia whereas their substrate (NADH and cyt *c*) affinity decreased (Lukyanova et al., 2008a). In the BC of HR rats, parameters of these enzymes remained unchanged or even decreased.

The physiological significance of such transformation may be that new isoforms of mitochondrial enzymes with new features emerge in the LR BC during adaptation. These features allow the enzymes to function in a broader range of their substrate (NADH or reduced cyt *c*) concentrations and at higher rates. The reduction degree of pyridine nucleotides and cytochromes, specifically cyt *c*, increases in hypoxia. Therefore, emergence of new kinetic properties in NADH cytochrome *c* reductase and cytochrome oxidase may provide more effective performance of these enzymes in long-term oxygen shortage. This may result in higher resistance of LR BC mitochondria to acute hypoxia.

Therefore, the economization of energy production characteristic of long-term adaptation to hypoxia is due to emergence of a new mitochondrial population with new properties, including the decreased content of respiratory transporters in the respiratory chain terminal region and the lower oxidizing capacity of these transporters, which, however, work more efficiently due to the increase deficiency of oxidative phosphorylation and the increased amount of mitochondria in the cell. On the whole, both these processes focus on replenishment of ATP losses, which should have occurred in these conditions.

However, in long-term adaptation to hypoxia, the significance of succinate oxidase oxidation gradually declines. In this process, the electron transport function of the NAD-dependent oxidation pathway gradually recovers, and the efficiency of C-I performance increases (Lukyanova et al., 2008a,b, 2009a). This may be related with the adaptation-induced emergence of new isoforms of the major complex I enzyme with new kinetic properties, which enhances the complex efficiency in high reduction of the pyridine nucleotide pool. Maintaining the high activity of C-II in these conditions may hamper the process.

Therefore, succinate contributes to formation of both immediate and delayed mechanisms of adaptation and resistance to hypoxia.

## Brain mitochondria and HIF-1 transcriptional activity in hypoxia

According to current concepts, the leading role in development of adaptation to hypoxia belongs to hypoxia-inducible factor 1 (HIF-1), a *specific protein factor induced by hypoxia*. This factor discovered in the early 1990s (Wang and Semenza, 1993; Semenza, 2002, 2007, 2009) functions as the major regulator of oxygen homeostasis. HIF-1 is a mechanism that the body uses to respond to hypoxia by controlling expression of proteins responsible for oxygen delivery to cells, i.e., HIF-1 mediates cell adaptive responses to changes in tissue oxygenation.

HIF-1 is a heterodimeric redox-sensitive protein consisting of two subunits, the cytoplasmic inducible, oxygen-sensitive **α** subunit (Semenza, 2002, 2007, 2009), which is expressed in practically all mammalian cells, and the constitutive **α** subunit. The HIF-1 activity depends primarily on the HIF-1α subunit whose synthesis is controlled by the MAPK and P13K signaling systems activated by the tyrosine kinase receptor. The receptor agonists include tyrosine hydroxylase, cytokines, growth factors (such as insulin-like factor), and succinate. Normally, the intracellular level of HIF-1α subunit is low because this subunit undergoes proteasomic degradation in oxygen-dependent reactions of prolyl hydroxylation and ubiquitination. Hypoxia creates prerequisites for inactivation of prolyl hydroxylase reactions and thereby provides HIF-1α stabilization and accumulation, induction of HIF-1α transcription and translocation to the nucleus, HIF-1α heterodimerization with the HIF1 β/ARNT subunit, formation of the active transcription complex HRE, expression of HIF-1 dependent target genes, and synthesis of protective, adaptive proteins (Semenza, 2002, 2007, 2009; Kim et al., 2006).

Our investigations have shown that under hypoxic preconditioning, neither free-radical processes nor cytokines and NO perform the function of signaling mechanisms for immediate adaptation responsible for the accumulation of HIF-1α in the early posthypoxic period, and they are likely to be only secondary messengers playing an important role in the formation of delayed adaptation (Kirova et al., 2013, 2014; Lukyanova, 2014).

At the same it is known that oxygen-dependent process of HIF-1α prolyl-hydroxylation and proteasomic degradation occurring in the cytosol of normoxic cells is coupled with utilization of the NAD-dependent substrate of TAC cycle, α-ketoglutarate, while another TAC cycle substrate, succinate, is an allosteric inhibitor of this process (Semenza, 2002, 2007, 2009; Hewitson et al., 2007). Hypoxia inhibits the malate-aspartate bypass, which provides α-ketoglutarate to the cytosol, whereas succinate synthesis is intensified. This creates prerequisites (along with O_2_ and Fe^2+^ shortage) for inactivation of prolyl hydroxylase reactions and HIF-1α stabilization, accumulation and potentiation of HIF-1α transcriptional activity.

Now it is proved that functioning of the mitochondrial respiratory chain is coupled with the hypoxia-induced transcriptional expression of HIF-1α. It was shown that even a partial (20%) suppression of C-II activity almost completely inhibited the hypoxic induction of HIF-1α. However it recovered in the presence of succinate (Vaux et al., 2001; Paddenberg et al., 2003; Napolitano et al., 2004; Selak et al., 2005; Hewitson et al., 2007; Koivunen et al., 2007).

**Figure 2:**
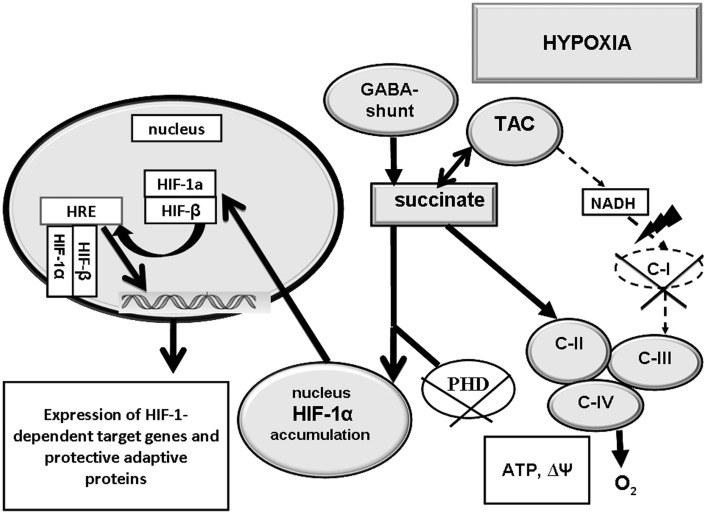
**Interaction of succinate oxidase-mediated oxidation (C-II) and HIF-1α transcriptional activity in hypoxia**. Activation of C-II contributes to inhibition of prolyl hydroxylase-mediated reactions (PHD) HIF-1α accumulation and translocation to the nucleus, and expression of HIF-1α-dependent adaptation genes. C-II, C-III, C-IV - mitochondrial enzyme complexes; TAC, tricarbonic acid cycle; PHD, prolyl hydroxylase-mediated reaction.

We have also shown, that induction of HIF-1α requires a low C-1 activity and a high C-II activity, i.e., potentiation of succinate oxidation (Lukyanova et al., 2008b, 2009b, 2011; Kirova et al., 2013, 2014; Lukyanova, 2014). If that is the case, a relationship should exist between activation of the succinate oxidase oxidation pathway and HIF-1α formation in hypoxia (Figure 2).

However, it should be kept in mind that excessive tissue accumulation of succinate in pathological conditions related with impairment of the SDH oxidative function or deficiency of this enzyme may result in excessively high tissue content of HIF-1α and, eventually, uncontrolled potentiation of proliferation, encephalomyopathy, and tumors (Chávez et al., 2000). Thus, succinate dehydrogenase mutations were shown to induce renal, gastric, and thyroid carcinoma, and degeneration of striatal spiny neurons (Huntington's disease) (Baysal, 2003; Selak et al., 2005).

## Brain expression of the succinate-dependent receptor, GPR91, in hypoxia

Hypoxic reprogramming and switching of the respiratory chain to the succinate oxidase oxidation pathway creates prerequisites for induction of another succinate oxidase signaling pathway, the expression of GPR91 receptor. In 2004, it was shown that succinate, a substrate for the TAC and SDH, the respiratory chain enzyme (C-II), was a specific ligand of GPR91, a metabotropic, purinergic, G protein-coupled receptor from the P2Y family localized in the plasma membrane (He et al., 2004). Succinate-stimulated expression of GPR91 is coupled with activation of Gq/11, G_o_ and G_i_ signaling pathways (He et al., 2004; Vargas et al., 2009). The Gq/11 subfamilies activate phospholipase Cβ, which converts phosphatidylinositol-4,5,-bisphosphate into inositol-1,4,5-trisphosphate and diacylglycerol (Fraser, 2008). Members of the Gi family, including, Go, activate a variety of phospholipases and phosphodiesterases, and promote opening of several ion channels. Gi family members can inhibit a subset of enzymes, thereby controlling the intracellular concentrations of cAMP (Hamm, 1998). Inhibition of Gi causes strong impairment of lymphocyte migration *in vitro* (Kaslow and Burns, 1992), suggesting that signaling through the Gi is involved in cell motility processes.

The succinate-dependent receptor, GPR91, is identified in more than 20 tissues (Stroka et al., 2001). The list of GPR91 localizations have been continuously expanding. The role of GPR91 is best studied for kidneys where the succinate-dependent expression of GPR91 involves the renin-angiotensin system and promotes development of renovascular hypertension, which is closely associated with atherosclerosis, diabetes, and renal insufficiency (He et al., 2004; Correa et al., 2007; Sadagopan et al., 2007; Sapieha et al., 2008; Toma et al., 2008; Vargas et al., 2009; Kermorvant-Duchemin et al., 2010; Deen and Robben, 2011). This activation is a part of a kidney-specific, paracrine signaling pathway initiated by high glucose concentrations. The GPR91 signaling cascade in kidneys includes local accumulation of succinate and expression and internalization of GPR91 in endothelial cells of kidney tubules. In the process of signal transduction, increased endothelial Ca^2+^ and formed NO and prostaglandin E2 exert a paracrine effect on adjacent renin-producing cells. This cascade can modulate renal function and facilitate elimination of metabolites by hyperfiltration.

The succinate-dependent GPR91 expression was found in the rodent retinal ganglion cell layer (Sapieha et al., 2008); in ischemic dendrite cells where GPR91 also functions as a trigger for Ca^2+^ mobilization and induction of proinflammatory cytokines (Rubic et al., 2008); in penumbra neurons and astrocytes of infracted brain where GPR91 stimulates an increase in microvessel density (Hamel et al., 2014); in hemopoietic progenitor cells where GPR91 stimulates their growth (Hakak et al., 2009). In hepatic ischemia, GPR91 is expressed only in hepatic stellate cells (Correa et al., 2007). In this case, the succinate-dependent effect is not related with hypertension. The receptor transforms the signal of increased extracellular succinate concentration into an intracellular signal, which provides activation of stellate cells in response to liver damage.

Information about the functional role of GPR91 is practically limited to these reports, which, however, do not answer the questions, what place the receptor occupies in the body response to hypoxia, and how GPR91 interacts with other signaling systems. According to our data, in the normoxic conditions, the succinate dependent receptor, GPR91, was present in all studied aerobic tissues. However GPR91 was absent in mitochondria-free red cells, which indirectly evidenced GPR91 dependence on mitochondria. The highest density of GPR91 was observed in the myocardium where the GPR91 density was 1.7 times higher than in BC and 2.7 times higher than in liver and kidneys (Lukyanova et al., accepted).

We have also shown that the *immediate* GPR91 expression response to a single hypoxic exposure is tissue-specific, depends on the intensity and duration of the hypoxic exposure, and its intensity does not correlate with the baseline receptor concentration. Thus, the baseline GPR91 concentration was the highest in the myocardium. However, the intensity of GPR91 expression induced by mild and moderate hypoxia was low and increased slowly. The GPR91 baseline concentration in the BC was half the myocardial concentration. Nevertheless, the GPR91 induction was observed in the BC in a broader range of low O_2_ in the inhaled air than in the myocardium and liver and was highly intensive (Lukyanova et al., accepted). These facts indicate higher reactivity and sensitivity to oxygen shortage of this succinate-dependent signaling system in the BC than in other studied organs, which may reflect a special significance of this system to the brain under the hypoxic conditions. F_*IO*2_ = 14% is a threshold value for GPR91 induction in the BC. In other tissues, GPR91 expression is not induced by these oxygen concentrations in inhaled air.

As distinct from a single, hypoxic session, repeated 60-min exposures (moderate hypoxia) induced a *phase of delayed* GPR91 expression in the BC, which formed regardless of whether the immediate phase of receptor expression was induced. The delayed phase developed following 5–8 sessions and reached a peak at 8–12 days. The delayed phase contemporized with development of delayed, non-specific tolerance of the body (Lukyanova et al., 2009a; Lukyanova et al., accepted). In none of the studied tissues, the phase of delayed GPR91 expression developed after a single hypoxic exposure.

Therefore, the succinate-dependent receptor, GPR91, is involved in the mechanism of formation of both immediate and delayed adaptation to hypoxia. The immediate GPR91 induction was the most intensive in hypoxic BC, which implied a high significance of this signaling pathway for the brain functioning. The fact that the phase of delayed GPR91 expression did not develop after a single hypoxic exposure and required repeated hypoxic sessions suggested that the mechanism of delayed phase formation did not depend on the immediate GPR91 expression but was mediated by other processes developing during a later phase.

**Figure 3:**
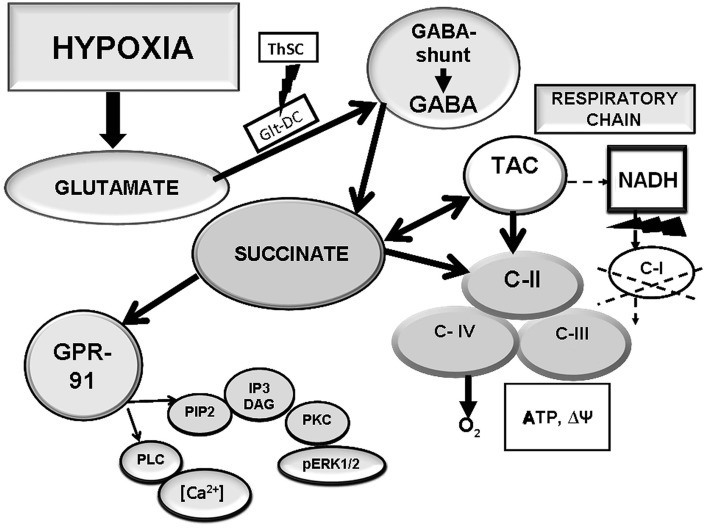
**Interaction of the respiratory chain and the GABA bypass aimed at formation of succinate, a GPR91 ligand, in hypoxic BC**.

The typical for BC immediate hypoxic expression of GPR91 may reflect a local increase in tissue succinate consistent with the hypoxic exposure and followed by succinate binding to the receptor as a paracrine signal. In the brain, the GABA bypass (Roberts cycle) may be a source of succinate in hypoxia. This brain-specific cycle consists of successive biochemical reactions activated by hypoxia/ischemia. In these reactions, gamma-aminobutyric acid (GABA) transforms to succinic acid, a C-II (SDH) substrate, through an intermediate step of succinic semialdehyde (Figure 3).

We have tested a possibility of using succinate formed in this cycle in the BC as a specific ligand for the GPR91 receptor. When the activity of glutamate decarboxylase, an enzyme responsible for GABA synthesis, modulation of the GABA bypass activity, and local endogenous formation of succinate, was inhibited with thiosemicarbazide (TSC), the immediate GPR91 expression was not observed in the BC under moderate hypoxia (Lukyanova et al., accepted). However, when TSC was administered together with succinate-containing agents (mexidol, proxipin, sodium succinate), the tissue receptor density recovered and was only slightly lower than the receptor density without the GABA inhibitor. These data demonstrate dependence of the GPR91 expression in the BC on succinate formed in hypoxia-induced reactions of the GABA bypass. This dependence, in its turn, suggests that in the BC, the induction of the succinate-dependent receptor, GPR91, performs a specific function in hypoxia. This function is related with the bypass performance and focused on elimination of glutamate excitotoxicity and maintenance of aerobic oxidation due to activation of the succinate-dependent pathway catalyzed by C-II. Therefore, the BC-specific immediate hypoxic expression of the succinate-dependent receptor, GPR91, is related with activity of the GABA bypass, which functions as a source of succinate both for the receptor and the respiratory chain.

## Pharmacological methods for protection of the brain respiratory chain in hypoxia

Prevalence of pathologies related with the disturbed function of C-I in both normoxic and hypoxic conditions, defines the exceptional importance and social significance of protecting the respiratory chain from oxygen shortage and protecting the body from the accompanying energy deficiency.

At present, two approaches to solving this problem exist—pharmacological and physical-therapeutic. The first approach (energotropic therapy) is based on the use of drugs containing an active substance that can interact with the mitochondrial respiratory chain and activate C-I bypassing oxidation pathways while the respiratory chain cytochrome region remains intact. This provides fast reversal of the energy deficiency induced by dysfunction of the NADH-dependent region.

The second approach (hypoxic therapy) is based on repeated exposures to mild hypoxia regimens (hypoxic preconditioning). These exposures induce a variety of adaptive responses, including formation of new isoforms of C-I enzymes with new redox properties, which allow C-I to function in the hypoxic conditions. Non-specific stress resistance of the body increases simultaneously.

The leading place among energotropic medicines belongs to succinate-containing drugs which have been successfully used as effective antihypoxic and adaptogenic compounds. The most effective of these drugs are structural derivatives of vitamin B_6_, which belong to 3-oxypyridine derivatives (mexidol, proxipin). These drugs are used in the early phase of acute disorders related with oxygen shortage (Luk'yanova et al., 1990; Kondrashova, 1993; Lukyanova and Dudchenko, 1999; Lukyanova et al., 2009a,b,c; Lukyanova, 2005; Nowak et al., 2008).

All succinate-containing drugs are very rapidly absorbed in the setting of different types of hypoxia/ischemia. They exert stabilizing or restoring effect on intracellular ATP as soon as in 15 min after administration. Studies of their mechanism showed that the respiratory chain uses succinate incorporated in the drug structure as an energy substrate. This process stimulates respiration and switches the electron flow from C-I to C-II (succinate monopolization of the respiratory chain proven by the increased respiration sensitivity to malonate and the reduced rotenone-sensitive respiration due to oxidation of NAD-dependent substrates) (Lukyanova and Dudchenko, 1999). Thus, succinate-containing drugs are succinate donors for the respiratory chain. In the hypoxic conditions, they function as antihypoxants potentiating activation of the succinate oxidase oxidation pathway to facilitate recovery and normalization of aerobic energy production (Lukyanova et al., 2009a).

Energotropic and antihypoxic effects of succinate-containing compounds are associated with (1) pronounced antioxidant properties; (2) modification and resynthesis of phospholipids, which decrease the membrane ionic permeability and K+ outflow from mitochondria by the concentration gradient; (3) normalizing effect on calcium metabolism; (4) catecholamine- mimetic, antiteratogenic, antitoxic, hepatoprotective, antiketogenic, and anticholesterolemic effects; (5) removal of excessive acetyl-Co-A associated with decreases in excessive lipids and their metabolites; (6) reduction and normalization of pH and elimination of metabolic acidosis (Kondrashova, 1993; Maevsky et al., 2001).

Along with the energotropic and antihypoxic effects, succinate-containing compounds beneficially influenced multiple vital functional parameters in the conditions of hypoxia and ischemia. These drugs reduced the death rate, recovered the body capability for gaining weight, decreased severity of neurological disorders and aggression typical for hypoxia, exerted antistress and normalizing effects on locomotor, exploratory, and emotional activities of animals (Luk'yanova et al., 1990; Lukyanova, 2005; Lukyanova et al., 2009a,b). Proxipin and mexidol are characterized by a broad range of pharmacological actions. In addition to pronounced antioxidant and psychotropic properties, they are able to increase resistance of the body to different types of hypoxia, reduce ATP losses in ischemic brain and myocardium, and normalize the process of oxidative phosphorylation (Lukyanova, 1997, 2005; Lukyanova and Dudchenko, 1999; Lukyanova et al., 2009b).

It should be also kept in mind that beneficial effects of succinate-containing drugs are supplemented with their modulating effects on the HIF-1α transcriptional activity and the coupled expression of genes for immediate and delayed adaptation, and involvement of critical intracellular signaling pathways via the succinate-dependent receptor, GPR91.

All this provides that the succinate therapy used in the early phase of acute disorders, i.e., during formation of immediate adaptive mechanisms (first 1–3 days of global cerebral ischemia, stroke, myocardial infarction, acute heart failure, traumatic shock, recovery after heart arrest, early postoperative period, after anesthesia, etc.,) exerts a pronounced protective, antihypoxic effect, and increases ATP in tissues.

## Conclusion

The data provided above show that the mitochondrial respiratory chain not only directily contribiutes to the development of both early and late adaptive responses in the conditions of hypoxia but is also involved in a sophisticated system of intra- and intercellular signaling, which provides formation of a systemic response to oxygen shortage.

**Figure 4:**
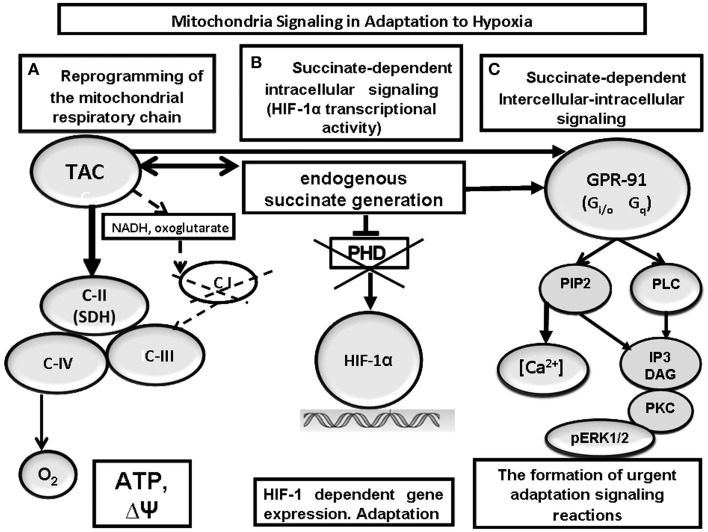
**Mitochondrial signaling in cell-to-cell interactions at hypoxia**. Reprogramming of the mitochondrial respiratory chain function in hypoxia is a regulatory and compensatory mechanism, which provides: (1) activation of succinate oxidation, a more efficient pathway of energy production in the conditions of rapidly progressing oxygen shortage **(A)**; (2) succinate-dependent PDH inhibition, subsequent stabilization of HIF-1α, and initiation of HIF-1α transcriptional activity **(B)**; (3) succinate-dependent activation of the GPR91 signaling pathway and related signaling pathways, stimulation of HIF-1α synthesis and transcriptional activity **(C)**.

The response to hypoxia initially mediated by C-I inhibition and C-II activation at the subcellular (mitochondrial) level triggers a cascade of succinate-dependent, interacting regulatory reactions, which develop at both cellular and systemic levels and regulate the maintenance of oxygen homeostasis in the body (Figure 4).

Transient, reversible, compensatory activation of respiratory chain complex II is a major mechanism of immediate adaptation to hypoxia necessary for (1) succinate-related energy synthesis in the conditions of oxygen deficiency and formation of urgent resistance in the body; (2) succinate-related stabilization of HIF-1α and initiation of its transcriptional activity related with formation of long-term adaptation; (3) succinate-related activation of the succinate-specific receptor, GPR91.

This mechanism participates in at least four critical regulatory functions: (1) *sensor* function related with changes in kinetic properties of C-I and C-II in response to a gradual decrease in ambient oxygen concentration; this function is designed for selection of the most efficient pathway for energy substrate oxidation in hypoxia; (2) *compensatory* function focused on formation of immediate adaptive responses to hypoxia and hypoxic resistance of the body; (3) *transcriptional* function focused on activated synthesis of HIF-1 and the genes providing long-term adaptation to low pO_2_; (4) *receptor* function, which reflects participation of mitochondria in the intercellular signaling system via the succinate-dependent receptor, GPR91. In all cases, the desired result is achieved by activation of the succinate-dependent oxidation pathway, which allows considering succinate as a signaling molecule.

Therefore the overall response of the body to oxygen shortage reflects a sophisticated, evolutionarily formed, multifunctional cell response, where energy metabolism performs a trigger, coordinating function of a protective signaling mechanism in the general hierarchy of intracellular processes.

Although succinate-dependent processes occur in different hypoxic tissues, in the BC these processes are observed in a greater range of low oxygen concentrations, more coordinated in time, and more intensive. This suggests, first, well-regulated endogenous, metabolic, anti-hypoxic defense, and adaptive capability of the brain and, second, a particular significance of brain adaptive processes for the development of tolerance in the whole body.

Analysis of bioenergetics mechanisms and related succinate-dependent signaling reactions in different hypoxia regimens showed that parameters of energy metabolism can be used as predictors and prognostic criteria for severity of hypoxic disorders.

The leading regulatory role of mitochondrial respiratory chain and succinate, its energy substrate, in the development of immediate adaptation to hypoxia suggests a possibility of modulating this process to optimize it. Practical medicine successfully uses various succinate-containing drugs, which exert beneficial effects in pathologies with a hypoxic component, due to their antihypoxic and energotropic effects.

### Conflict of interest statement

The authors declare that the research was conducted in the absence of any commercial or financial relationships that could be construed as a potential conflict of interest.

## Footnotes

^1^Any population of animals is known to contain animals with dissimilar tolerance to hypoxia, which correlates with features of the higher nervous system and the functional metabolic profile (Lukyanova, 1997; Lukyanova et al., 2008b, 2009a,b). This dissimilarity is based on differences in the state of the central nervous system, neuro-humoral regulation, stress-activating, and stress-limiting systems, oxygen transporting function of blood, and the state of membranes and receptors. Low-resistant (LR) animals are considered to have a weak type of the nervous system, increased excitability and emotional reactivity, less developed internal inhibition, and high exhaustibility of excitation. LR animals respond to hypoxia with agitation and high locomotor activity. In LR rats, acute hypoxia results in a greater variety of functional and metabolic parameters than in high-resistant (HR) rats. In contrast, HR to hypoxia animals have reduced excitability and anxiety, milder aggressiveness, more pronounced internal inhibition, low sensitivity to any provocative factors, and tendency to social domination. Acute hypoxia induces an inhibitory response in HR rats (Goryacheva et al., 1993; Livanova et al., 1992; Lukyanova, 1997, 2005; Lukyanova et al., 2009c).

## Abbreviations

ATP: adenosine triphosphate
ADP: adenosine diphosphate
BC: brain cortex
C-I, II, III, IV: mitochondrial complexes I, II, III, IV
GABA: gamma-aminobutyric acid
GPR91: succinate-related guanine nucleotide - binding protein-coupled receptor
HBH: hypobaric hypoxia
HIF: hypoxia-inducible factor
HRE: hypoxia response element
HR: high-resistance rats
LR: low-resistance rats
NAD, NADH: nicotine adenine nucleotides
PC: phosphocreatine
PDH – HIF: prolyl-hydroxylase
PDK: pyruvate dehydrogenase kinase
SDH: succinate dehydrogenase.
